# Acquisition of EGFR TKI resistance and EMT phenotype is linked with activation of IGF1R/NF-κB pathway in EGFR-mutant NSCLC

**DOI:** 10.18632/oncotarget.21170

**Published:** 2017-09-21

**Authors:** Ling Li, Xiajing Gu, Jinnan Yue, Qingnan Zhao, Dacheng Lv, Hongzhuan Chen, Lu Xu

**Affiliations:** ^1^ Department of Pharmacology, Shanghai Jiao Tong University School of Medicine, Shanghai, China

**Keywords:** EMT, IGF1R, NF-κB, NSCLC, EGFR TKI resistance

## Abstract

Epithelial-mesenchymal transition (EMT) is clinically associated with acquired resistance to epidermal growth factor receptor (EGFR) tyrosine kinase inhibitors (TKI) in non-small cell lung cancers (NSCLC). However, the mechanisms promoting EMT in EGFR TKI-resistant NSCLC have not been fully elucidated. Previous studies have suggested that IGF1R signaling is involved in both acquired EGFR TKI resistance in NSCLC and induction of EMT in some types of tumor. In this study, we further explored the role of the IGF1R signaling in the acquisition of EMT phenotype associated with EGFR TKI resistance in mutant-EGFR NSCLC. Compared to gefitinib-sensitive parental cells, gefitinib-resistant (GR) cells displayed an EMT phenotype associated with increased migration and invasion abilities with the concomitant activation of IGF1R and NF-κB p65 signaling. Inhibition of IGF1R or p65 using pharmacological inhibitor or specific siRNA partially restored sensitivity to gefitinib with the concomitant reversal of EMT in GR cells. Conversely, exogenous IGF1 induced both gefitinib resistance and accompanying EMT in parental cells. We also demonstrated that IGF1R could phosphorylate downstream Akt and Erk to activate NF-κB p65. Taken together, our findings indicate that activation of IGF1R/Akt/Erk/NF-κB signaling is linked to the acquisition of EGFR TKI resistance and EMT phenotype in EGFR-mutant NSCLC and could be a novel therapeutic target for advanced NSCLC.

## INTRODUCTION

In the last decade, the discovery of activating EGFR (epidermal growth factor receptor) mutations in NSCLC has shifted the treatment paradigm to molecular targeted therapies. EGFR tyrosine kinase inhibitors (TKI) have become the first-line choice in NSCLC patients harboring EGFR activating mutations. However, despite of the initial dramatic response, all patients inevitably develop acquired resistance which is mediated by the emergence of secondary EGFR T790M mutation or amplification of MET gene in approximately 55% and 5% of patients, respectively [1–5].

Emerging evidence has shown that epithelial-mesenchymal transition (EMT), which is an evolutionarily conserved process, is implicated in both tumor progression and resistance to therapy [6–10]. The EMT is a reversible process in which epithelial cells lose cell-cell adhesion and cell polarity, decrease the expression of epithelial cells marker such as E-cadherin, increase the expression of mesenchymal cell markers such as vimentin, fibronectin and N-cadherin, as well as increase the activity of matrix metalloproteinases (MMPs). These alterations lead to an increase in fibroblastoid and mesenchymal morphology, invasiveness and resistance to apoptosis. The association between EMT and local invasion and dissemination at distant organs has been demonstrated in numerous *in vivo* and *in vitro* studies [6–8]. Moreover, many studies have shown that EMT confers resistance of cancer cells to chemotherapy, targeted therapy and radiation therapy [5, 9, 10]. In NSCLC, several studies have reported that acquisition of EMT is associated with EGFR TKI resistance in EGFR-mutant cell lines and tumor specimens [11–15]. Since EMT is known to be reversible, hopefully, strategies to prevent or reverse EMT could improve outcomes for advanced cancer patients [16, 17].

EMT is an extremely well-organized process and induction of EMT seems to be cell or tissue specific and requires the cooperation of multiple signaling pathways and regulators. The molecules involved in EMT process could be classified into three groups: the effectors executing EMT such as E-cadherin, the transcription factors acting as regulators to orchestrate EMT such as Snail, Slug, Zeb and Twist families, and extracellular inducers that engage the cells in EMT, such as TGFβ and the growth factor families [6, 18]. Emerging evidence has demonstrated that those pathways are exploited by treatment-resistant tumor cells to acquire EMT phenotype in several tumor types, such as head and neck squamous cell carcinoma [19], prostate cancer [20], breast cancer [21, 22], pancreatic cancer [23], ovarian carcinoma [24], hepatocellular carcinoma [25], gastric cancer [26], glioblastoma [27] and lung cancer [28–33]. However, the mechanisms promoting EMT in EGFR TKI-resistant NSCLC cells have not been fully elucidated.

IGF1R (Insulin-like growth factor 1 receptor) activation has been reported to confer acquired resistance to EGFR TKI [34–41]. IGF1R activates EGFR downstream signaling including PI3K/AKT and MAPK/ERK pathways to bypass the inhibited EGFR. Interestingly, few studies have shown that IGF1R pathway is also involved in the acquisition of EMT phenotype in some types of cancer cells, such as prostate cancer [42], breast cancer [43, 44] and lung cancer [45]. However, the underlying mechanism remains unclear. In the present study, we demonstrated that activation of IGF1R was implicated in acquired resistance to gefitinib in EGFR-mutant NSCLC, which is consistent with previous studies [34–41]. More importantly, we found that acquisition of EMT phenotype in those resistant cells was mediated by IGF1R/AKT/ERK/NF-κB signaling pathway. Evidence has indicated that NF-κB activation can upregulate the expression of Snail, Slug and ZEB1/2 and is required for the induction and maintenance of EMT in several cancer types [46–50]. To our best knowledge, this is the first study to demonstrate that NF-κB is involved in the acquisition of EMT phenotype in gefitinib-resistant EGFR-mutant NSCLC. Furthermore, we found that inhibition of IGF1R or NF-κB p65 led to reversal of EMT phenotype with the concomitant partial restoration of sensitivity to gefitinib, suggesting that IGF1R/NF-κB pathway could be a novel target for treatment of advanced NSCLC patients.

## RESULTS

### Establishment of gefitinib-resistant (GR) lung adenocarcinoma cell lines and validation of EGFR TKI resistance

**Figure 1:**
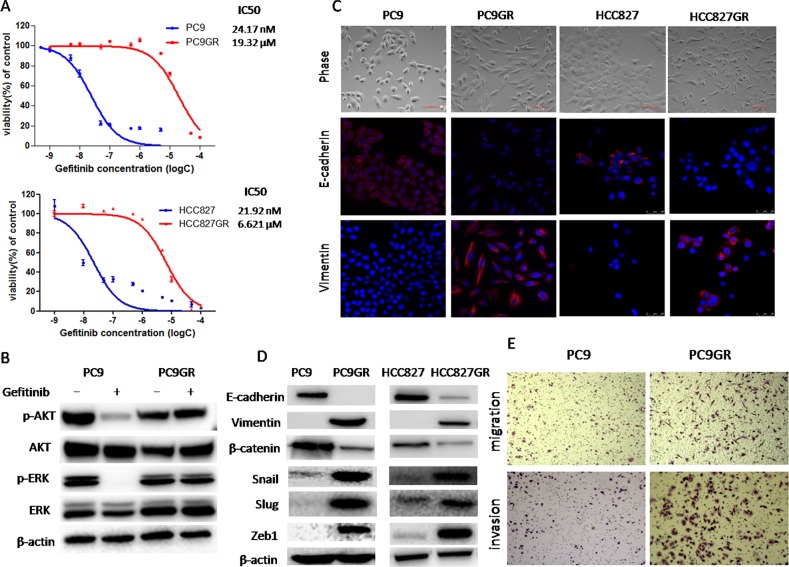
Gefitinib-resistance (GR) cells acquired an epithelial-mesenchymal transition (EMT) phenotype and increased migration and invasion abilities **(A)** The sensitivity to gefitinib of PC9GR, HCC827GR and their respective parental cells was examined by CCK8 assay. Cells were treated with the indicated concentration of gefitinib for 72h. IC50 values were calculated and marked for each curve. **(B)** The expression of p-Akt, Akt, p-Erk and Erk of PC9 and PC9GR cells treated with 20nM gefitinib for 2h was measured by Western blot. **(C)** Morphology of GR and parental cells. Photographs were taken at × 40 magnification. Scale bar: 100μm. The expression of E-cadherin and vimentin was analyzed by immunofluorescence staining. Nuclei were visualized with DAPI staining. **(D)** The expression of E-cadherin, vimentin, β-catenin, Snail, Slug and ZEB1 of GR and parental cells was examined by Western blotting. **(E)** Migration and invasion abilities of PC9 and PC9GR cells were examined by transwell assays. The cells were incubated for 8h (for migration) or 24h (for invasion). Those migrated cells remaining on the bottom surface were fixed, stained and photographed. Photographs were taken at ×40 magnification.

To explore the mechanisms of acquired EGFR TKI resistance in NSCLC, we established the gefitinib-resistant (GR) lung adenocarcinoma cells. EGFR-mutant NSCLC cell lines, PC9 and HCC827, were exposed to increasing concentrations of gefitinib for more than 6 months. As shown in Figure 1A, PC9GR and HCC827GR cells were highly insensitive to gefitinib, with a 300∼800-fold increase in IC50 compared to their respective parental cells. Next, in PC9 and PC9GR cells, we evaluated the effects of gefitinib on the phosphorylation of Akt and Erk1/2 which are two major downstream pathways of EGFR activation. Western blot demonstrated that when treated with 20nM gefitinib for 2h, PC9GR cells showed no significant change in either Akt or Erk phosphorylation whereas PC9 cells showed a dramatic inhibition of both Akt and Erk phosphorylation (Figure 1B), suggesting the existence of a bypass pathway to activate EGFR downstream signaling in GR cells. No T790M mutation or MET gene amplification was detected in either resistant cell lines (data not shown).

### GR cells acquired EMT phenotype and had increased migratory and invasive abilities

As shown in Figure 1C, phase-contrast photo-micrographs of cultured GR cells had shown remarkable morphologic changes consistent with EMT compared to their respective parental cells. GR cells displayed elongated, fibroblastoid morphology, whereas parental cells had an epithelial cobblestone appearance and a rounded shape. To confirm that whether GR cells underwent EMT, we examined the expression of EMT markers using Western blotting analysis. We found that the expression of epithelial markers such as E-cadherin and β-catenin was significantly decreased in GR cells, whereas the expression of mesenchymal markers such as vimentin, Snail, Slug and Zeb1 was highly elevated in GR cells (Figure 1D). Furthermore, immunofluorescence staining was also used to examine the expression of E-cadherin and vimentin. As shown in Figure 1C, parental PC9 and HCC827 cells displayed typical epithelial staining pattern of E-cadherin that was predominantly located on the surface of cells while PC9GR and HCC827GR cells exhibited robust staining of vimentin that was typically observed in the cytoplasm of mesenchymal cells. Taken together, these results demonstrated that GR cells acquired EMT phenotype, which could be involved in acquired EGFR TKI resistance.

It has been well established that induction of EMT was associated with aggressive biological behavior, such as increased migration and invasion [6–8]. To assess whether the acquisition of EMT phenotype of GR cells correlates with functional changes, we evaluated *in vitro* migration and invasion ability using transwell migration and invasion assays, respectively. As shown in Figure 1E and Supplementary Figure 3A, the number of PC9GR cells migrating through transwell chamber in both non-basement membrane chamber (migration assay) and matrigel-coated chamber (invasion assay) was dramatically increased than that of PC9 cells, demonstrating that PC9GR cells had increased migration and invasion capabilities.

### IGF1R/NF-κB p65 signaling was activated in GR cells

**Figure 2:**
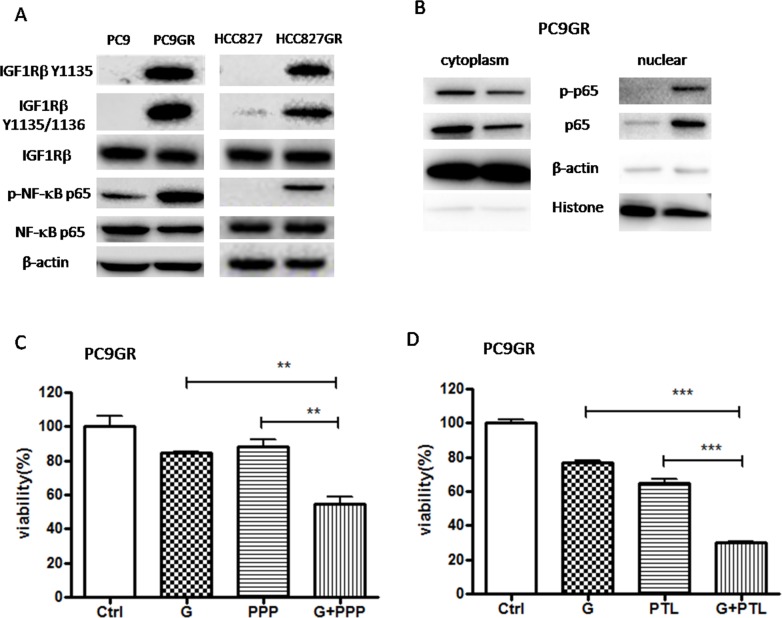
IGF1R/NF-κB p65 signaling was activated in GR cells and inhibition of IGF1R/NF-κB p65 increased the sensitivity of GR cells to gefitinib **(A)** The expression of IGF1Rβ, p-IGF1Rβ, NF-κB p65, p-p65 of GR and parental cells was examined by Western blotting. **(B)** The expression of p-p65, p65 in cytoplasmic extracts or nuclear extracts of PC9GR cells was measured by Western blot. β-actin and H3 histone was used as loading control for cytoplasmic extracts and nuclear extracts, respectively. **(C)** and **(D)** The sensitivity to 5μM gefitinib of PC9GR cells pre-treated with 0.1μM PPP (C) or 1μM PTL (D) was examined by CCK8 assay. Cells were pre-treated with 0.1μM PPP (C) or 1μM PTL (D) for 2h and then subjected to 5μM gefitinib for 72h. The data are presented as the means ± SEM and normalized to control cells treated with solvent (DMSO). ^*^ P < 0.05; ^**^ P < 0.01; ^***^ P < 0.001, compared with control.

NF-κB has been identified as a critical mediator of EMT in cancer progression [46–50] and IGF1R pathway has been reported to be involved in EGFR TKI resistance and induction of EMT in lung cancer [34–41, 45]. To determine whether IGF1R or NF-κB signaling is involved in GR cells, we investigated the expression and activation of IGF1R and NF-κB p65 using Western blot. As shown in Figure 2A, the expression of total IGF1R and NF-κB p65 has no change while the expression of phosphorylated IGF1R (Ser1135/1136) and NF-κB p65 (Ser536) protein was elevated in both GR cells compared to their respective parental cells, suggesting that IGF1R and NF-κB p65 signaling were activated in both GR cells. Moreover, we isolated cytoplasmic and nuclear fractions to examine the subcellular localization of p65. As show in Figure 2B, the nuclear p65 and p-p65 were remarkably increased while the cytoplasmic p65 and p-p65 were decreased in PC9GR cells compared to PC9 cells, suggesting that the nuclear translocation of p65 and p-p65 was upregulated in GR cells. Taken together, our result showed that in GR cells the IGF1R/NF-κB p65 signaling was activated.

To validate the activation of IGF1R/NF-κB p65 signaling in gefitinib-resistant mouse lung adenocarcinoma, we used genetically engineered mice harboring a doxycycline-inducible human EGFR exon 19 deletion transgene (hEGFR Del19) [51]. The mice were put on continuous doxycycline diets at 6 weeks of age until sacrificed. Tumor-bearing mice were treated orally with gefitinib daily for the first two weeks and then every other day for at least 4 month until persistent symptoms of respiratory distress emerged as previously reported [52]. Then, those mice were sacrificed and tumor nodules from lungs were collected for analysis. As shown in Supplementary Figure 1, we found that EMT was observed in tumors harvested from one long-term gefitinib-treated mouse (relapse tumor) out of 11 with substantially increased expression of mesenchymal markers such as vimentin, Zeb1 and Snail compared to tumors harvested from vehicle-treated littermate control mouse (primary tumor). These results were consistent with previous study in which only two out of 37 relapsed patients showed EMT in re-biopsies [5]. More importantly, we also found that this particular tumor had significantly increased expression of total IGF1R, p-IGF1R, total p65 and p-p65. Taken together, our results indicated that IGF1R/NF-κB p65 signaling was activated and appeared to be associated with acquisition of EMT phenotype in gefitinib-resistant mouse lung tumor tissues.

### Inhibition of IGF1R/NF-κB p65 signaling partially restored the sensitivity of PC9GR cells to gefitinib

To determine whether the inhibition of IGF1R/NF-κB pathway might re-sensitize PC9GR cells to gefitinib, we performed cell viability assay on PC9GR cells treated with 5μM gefitinib (G) for 72h (Figure 2C and 2D). Our results showed that pre-treatment with IGF1R inhibitor picropodophyllin (PPP) or NF-κB inhibitor parthenolide (PTL) significantly increased the sensitivity of PC9GR cells to gefitinib, suggesting that IGF1R/NF-κB activation was involved in the acquisition of gefitinib resistance in EGFR-mutant NSCLC.

### Inactivation of IGF1R/NF-κB p65 reversed EMT and suppressed migration and invasion abilities in PC9GR cells

**Figure 3:**
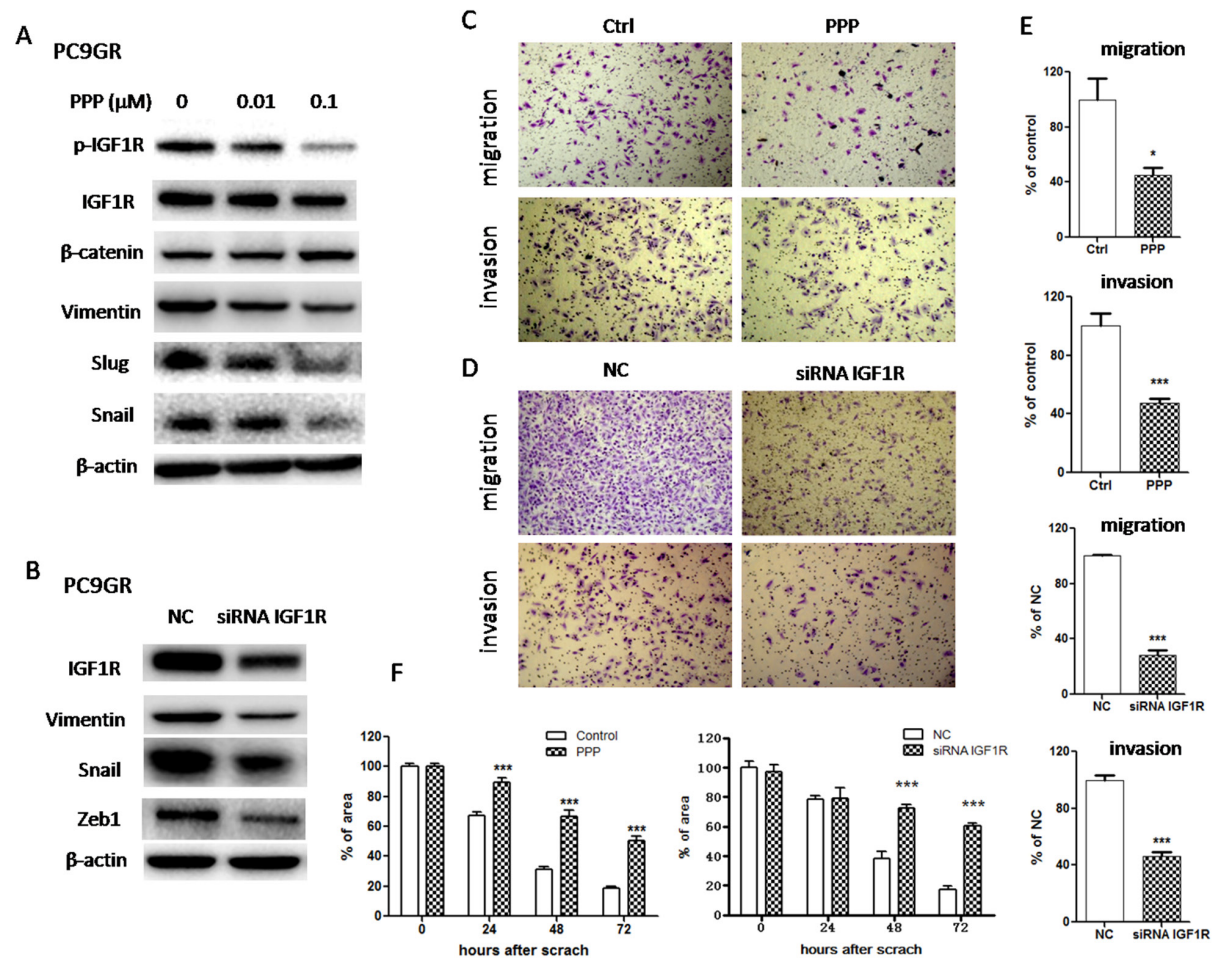
Inhibition of IGF1R reversed EMT and suppressed migration and invasion in PC9GR cells **(A)** The expression of IGF1R, p-IGF1R, β-catenin, vimentin, Snail and Slug of PC9GR cells treated with indicated concentrations of PPP for 72h was examined by Western blotting. **(B)** The expression of IGF1R, vimentin, Snail and Zeb1 of PC9GR cells transfected with siRNA IGF1R for 72h was examined by Western blotting. **(C)** and **(D)** Migration and invasion abilities of PC9GR cells treated with 0.1μM PPP (C) or transfected with siRNA IGF1R (D) were examined by transwell assays. The cells were incubated for 8h (for migration) or 24h (for invasion). Those migrated cells remaining on the bottom surface were fixed, stained, photographed and counted. Photographs were taken at ×40 magnification. **(E)** Quantification of transwell migration and invasion assays (C and D). The number of cells was counted from at least four independent microscopic fields. **(F)** Quantification of wound healing assay of PC9GR cells treated with 0.1μM PPP or transfected with siRNA IGF1R. The open wound area was quantified by ImageJ from at least four independent microscopic fields and normalized to the area at time 0. The data are presented as the means ± SEM and normalized to control cells treated with solvent (DMSO) or transfected with siRNA NC. ^*^ P < 0.05; ^**^ P < 0.01; ^***^ P < 0.001, compared with control.

**Figure 4:**
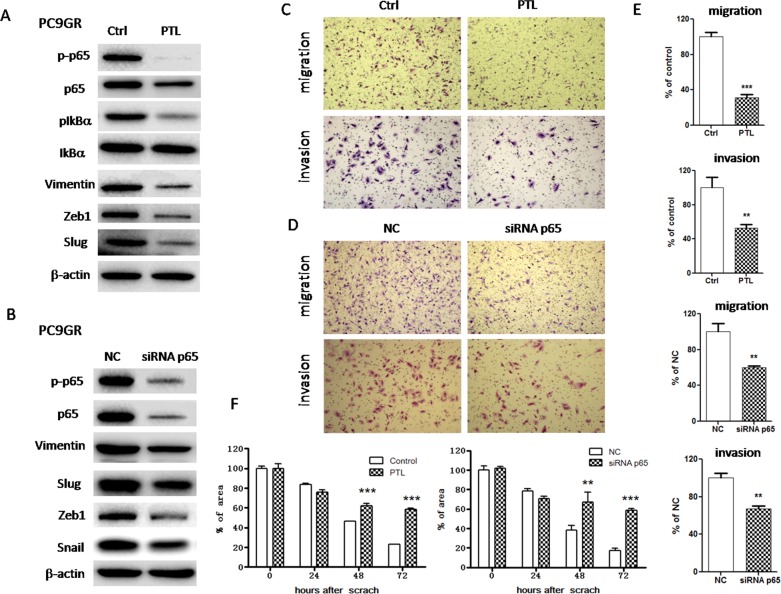
Inhibition of NF-κB p65 reversed EMT and suppressed migration and invasion in PC9GR cells **(A)** The expression of p65, p-p65, IkBa, p-IkBa, vimentin, Slug and Zeb1 of PC9GR cells treated with 1μM PTL for 72h was examined by Western blotting. **(B)** The expression of p65, p-p65, vimentin, Snail, Slug and Zeb1 of PC9GR cells transfected with siRNA p65 for 72h was examined by Western blotting. **(C)** and **(D)** Migration and invasion abilities of PC9GR cells treated with 1μM PTL (C) or transfected with siRNA p65 (D) were examined by transwell assays. The cells were incubated for 8h (for migration) or 24h (for invasion). Those migrated cells remaining on the bottom surface were fixed, stained, photographed and counted. Photographs were taken at ×40 magnification. **(E)** Quantification of transwell migration and invasion assays (C and D). The number of cells was counted from at least four independent microscopic fields. **(F)** Quantification of wound healing assay of PC9GR cells treated with 1μM PTL or transfected with siRNA p65. The open wound area was quantified by ImageJ from at least four independent microscopic fields and normalized to the area at time 0. The data are presented as the means ± SEM and normalized to control cells treated with solvent (DMSO) or transfected with siRNA NC. ^*^ P < 0.05; ^**^ P < 0.01; ^***^ P < 0.001, compared with control.

To determine whether IGF1R/NF-κB activation might be involved in the acquisition of EMT phenotype and increased migration and invasion abilities in GR cells, we treated PC9GR cells with either pharmacological inhibitor or specific siRNA to inhibit IGF1R or p65. As shown in Figure 3A, IGF1R inhibitor PPP decreased the expression of mesenchymal markers such as vimentin, Snail and Slug in a dose-dependent manner. Moreover, siRNA IGF1R also decreased the expression of vimentin, Snail and Zeb1 (Figure 3B). These results suggested that EMT was reversed. Meanwhile, PPP or siRNA IGF1R decreased *in vitro* migration and invasion abilities of PC9GR cells, using wound healing assay (Figure 3F and Supplementary Figure 2) and transwell migration and invasion assays (Figure 3C-3E). Furthermore, inhibition of p65 using either PTL or siRNA p65 also reversed EMT (Figure 4A and 4B) and simultaneously decreased *in vitro* migration and invasion capabilities in PC9GR cells (Figure 4C-4F and Supplementary Figure 2).

### IGF1-mediated IGF1R activation attenuated the sensitivity to gefitinib and induced EMT in parental PC9 cells

**Figure 5:**
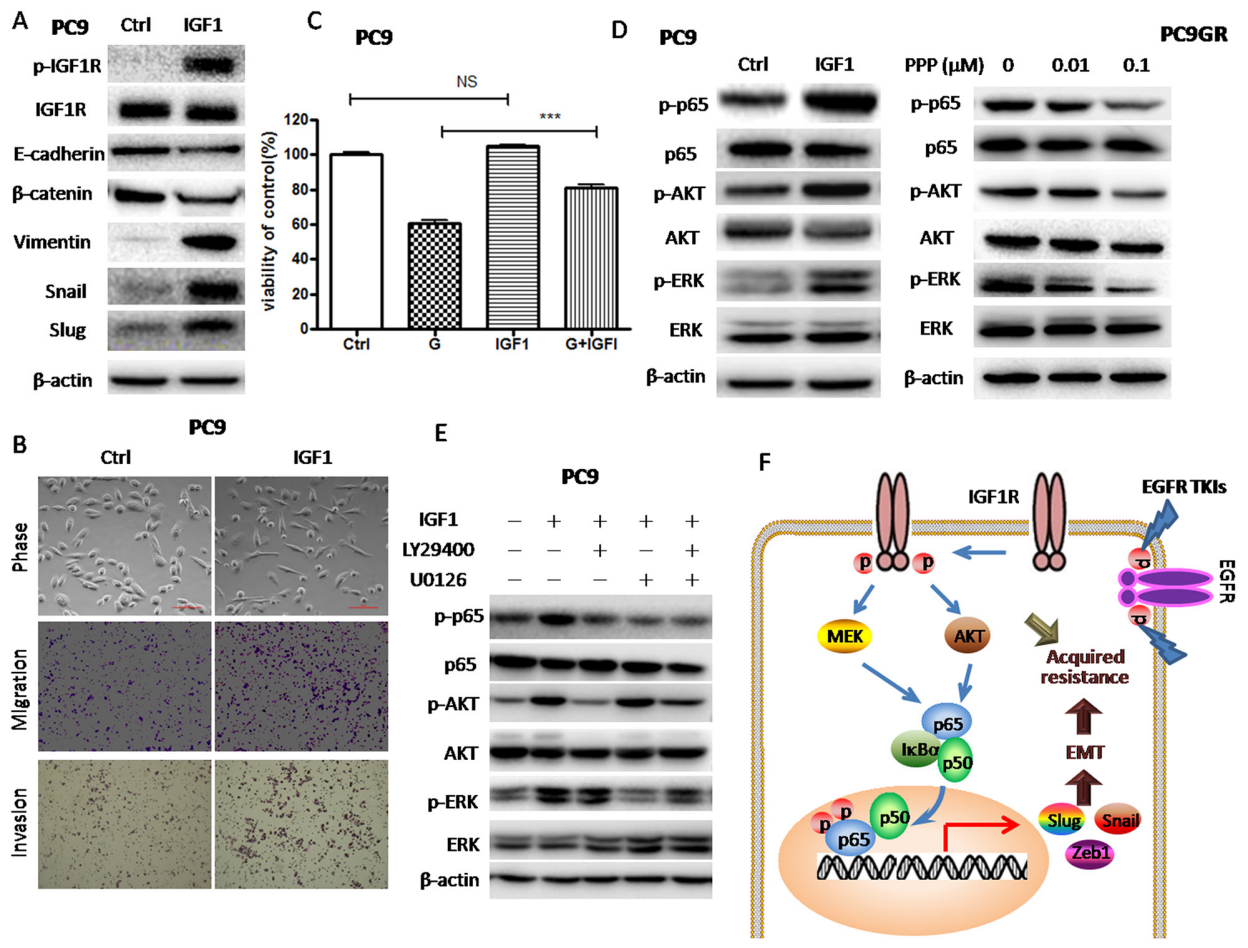
IGF1R activation by exogenous IGF1 led to Akt/Erk/NF-κB p65 activation, decreased sensitivity to gefitinib and EMT induction **(A)** The expression of IGF1R, p-IGF1R, E-cadherin, β-catenin, vimentin, Snail and Slug of PC9 cells treated with 100ng/ml IGF1 for 72h was examined by Western blotting. **(B)** Morphology of PC9 cells treated with 100ng/ml IGF1 for 72h. Migration and invasion abilities of PC9 cells treated with 100ng/ml IGF1 were examined by transwell assays. Photographs were taken at ×40 magnification. Scale bar: 100μm. The number of cells was counted from at least four independent microscopic fields. **(C)** The sensitivity to 20nM gefitinib of PC9 cells pre-treated with 100ng/ml IGF1 was examined by CCK8 assay. PC9 cells were pre-treated with 100ng/ml IGF1 for 2h and then subjected to 20nM gefitinib for 72h. **(D)** The expression of p65, p-p65, p-Akt, Akt, p-Erk and Erk of PC9 cells treated with 100ng/ml IGF1 for 72h or PC9GR cells treated with indicated concentrations of PPP for 72h was examined by Western blotting. **(E)** The expression of p65, p-p65, p-Akt, Akt, p-Erk and Erk of PC9 cells treated with 100ng/ml IGF1 for 72h was examined by Western blotting. PC9 cells were pre-treated with 25μM LY294002 and 5μM U0126 alone or in combination and then subjected to 100ng/ml IGF1 for 72h. **(F)** A proposed model for IGF1R/Akt/Erk/NF-κB p65 signaling pathways in the acquisition of EMT phenotype of gefitinib-resistant NSCLC. The data are presented as the means ± SEM and normalized to control cells treated with solvent (DMSO). ^*^ P < 0.05; ^**^ P < 0.01; ^***^ P < 0.001, compared with control.

To test whether IGF1R activation can induce EMT and conferred gefitinib resistance at the same time, we treated parental PC9 cells with exogenous IGF1 to activated IGF1R. First, Western blot showed that IGF1 increased the expression of phosphorylated IGF1R indicating IGF1R was activated (Figure 5A). Then, the expression of EMT markers was examined by Western blot. As shown in Figure 5A, the expression of epithelial markers such as E-cadherin and β-catenin was significantly decreased while the expression of mesenchymal markers such as vimentin, Snail and Slug was highly elevated upon IGF1 treatment, suggesting that IGF1R activation by IGF1 induced EMT in PC9 cells. Phase-contrast photomicrographs of PC9 cells treated with IGF1 also showed EMT phenotype (Figure 5B). Moreover, transwell assays showed that IGF1 increased *in vitro* migration and invasion abilities in PC9 cells (Figure 5B and Supplementary Figure 3B). To determine whether the activation of IGF1R might de-sensitize PC9 cells to gefitinib, we performed cell viability assay on PC9 cells treated with 20nM gefitinib (G) for 72h. The results showed that IGF1 alone had no effect on PC9 cell proliferation but de-sensitized PC9 cells to gefitinib (Figure 5C). Those results suggested that IGF1R activation was involved in the acquisition of both gefitinib resistance and EMT phenotype in GR cells.

### Activation of NF-κB p65 was mediated by IGF1R-induced Akt and Erk phosphorylation

To clarify the molecular link between IGF1R and NF-κB p65, we treated parental PC9 cells with exogenous IGF1 to activate IGF1R and PC9GR cells with PPP to inhibit IGF1R, respectively. As shown in Figure 5D, IGF1 increased the phosphorylation of p65 as well as the phosphorylation of Akt and Erk in PC9 cells, while PPP decreased the phosphorylation of p65 as well as the phosphorylation of Akt and Erk in PC9GR cells. Those results suggested that IGF1R-induced p65 activation was mediated by Akt and Erk. Furthermore, we pre-treated PC9 cells with LY29400 and U0126 to inhibit Akt and Erk phosphorylation. As expected, LY29400 and U0126 alone or in combination could inhibit IGF1R-induced p65 phosphorylation (Figure 5E). Taken together, Akt and Erk phosphorylation induced by IGF1R activation could activate NF-κB p65 in GR cells.

## DISCUSSION

Development of acquired EGFR TKI resistance remains the major therapeutic barrier in NSCLC. To identify novel acquired resistance mechanisms, previous studies had established *in vitro* model of acquired EGFR TKI-resistant sublines by exposing sensitive parental cell lines to increasing dosages of EGFR TKI [4, 38, 53–58]. In the present study, PC9 and HCC827 cells, both of which harbor the mutant EGFR, were used to establish gefitinib-resistant sublines (PC9GR and HCC827GR) by continuously culturing in gefitinib. We observed that in both gefitinib-resistant sublines, neither T790M nor MET amplification was detected. In some previously published studies, consistent with our results, there was neither T790M nor MET amplification detected in EGFR TKI-resistant sublines [53–56]. In other studies, either T790M or MET amplification was detected in resistant sublines [4, 38, 56–58]. The reasons for this discrepancy are not well understood but could be due to intratumoral heterogeneity among cell lines, differences in culturing conditions or treatment regimen, sensitivity of detection method et al [56, 58].

A growing body of evidence strongly suggests that there are intricate links between EMT and EGFR TKI resistance [5, 9–15]. For instance, Sequist et al studied re-biopsies taken from 37 EGFR-mutated patients treated with erlotinib or gefitinib at the time that drug resistance was acquired. EMT was observed in two cases of acquired TKI resistance [5]. Due to the general lack of tumor biopsy material, it is difficult to estimate the occurrence of EMT. In contrast to the few investigations of EMT as an EGFR TKI acquired resistance mechanism in NSCLC patients, there are some cell studies showing that EGFR TKI treatment could select cells capable of bypassing signaling through activating of RTKs such as IGF1R, FGFR1 or TGFβ and co-occurring induction of EMT could generate mesenchymal-like cells which has survival or anti-apoptotic advantage [29, 30, 33]. However, the molecular mechanism for EMT induction related to EGFR TKI resistance is not entirely clear yet. In the present study, we observed that both gefitinib-resistant sublines acquired EMT phenotype and had increased migration and invasion capacities, which are consistent with previous studies [45]. On the other hand, we treated parental PC9 cells, which are sensitive to gefitinib, with exogenous IGF1 and observed a decreased sensitivity to gefitinib with the concomitant induction of EMT. These data suggested that there was a link between EGFR TKI resistance and EMT. However, the underlying mechanisms remain unclear.

Many of signaling pathways, including TGF-β, NF-κB and growth factor signaling pathways, are found to be critical for EMT induction. Previous studies have shown that IGF1R activation can induce EMT as well as contribute to the acquired EGFR TKI resistance. For example, IGF1R phosphorylation was found increased in EGFR TKI-resistant NSCLC cells [34–37] and activation of IGF1R decreased the sensitivity of NSCLC cells to EGFR TKI [45]. IGF1R activation could also promote EMT in some types of cancer cells [41–45]. Consistent with these studies, we found that both gefitinib-resistant NSCLC cells and gefitinib-resistant mouse lung tumor tissues acquired EMT phenotype along with IGF1R activation. However, EMT was observed in resistant tumors from only one mouse, which is the limitation of our *in vivo* study. To gain further insight whether IGF1R could regulate EMT in gefitinib-resistant cells, we inhibited IGF1R activity by IGF1R inhibitor PPP and found that EMT was reversed and migration and invasion abilities were reduced in these GR cells treated with PPP, along with increased sensitivity to gefitinib. Moreover, we downregulated the expression of IGF1R by siRNA in GR cells and found that IGF1R siRNA also reversed EMT and reduced migration and invasion abilities. Exogenous administration of IGF1 to PC9 cells induced EMT as well as decreased sensitivity to gefitinib through IGF1R activation. Taken together, these results suggested that activation of IGF1R could result in EGFR TKI resistance and mediate the induction of EMT phenotype which could also contribute to EGFR TKI resistance. Therefore, IGF1R seems to be a promising target in treating advanced NSCLC patients. Unfortunately, clinical trials failed to show benefit from targeting IGF1R in treating advanced NSCLC patients, partially due to the lack of biomarkers to select patient populations [59–62].

However, our results raised the question of how the activation of IGF1R induces EMT in GR cells and whether IGF1R signaling crosstalks with other signaling pathways critical to EMT induction, such as NF-κB p65 signaling pathway. First, we examined the expression and activation of NF-κB p65 and found that both total p65 and phospho-p65 were remarkably increased in nucleus in GR cells, suggesting that NF-κB was activated in GR cells. In addition, we found that the expression of both total p65 and phospho-p65 was dramatically increased in gefitinib-resistant mouse lung tumor tissues with EMT phenotype. The activation of NF-κB is known to upregulate the expression of EMT transcription factors such as Snail, Slug and Zeb1 resulting in the induction of EMT [46–50], which is consistent with our findings. Then, we inhibited NF-κB p65 activation by parthenolide (PTL) in GR cells as previously described [63] and found EMT was reversed and migration and invasion abilities were reduced. And more importantly, the sensitivity to gefitinib was also increased in these GR cells treated with PTL. Furthermore, we downregulated the expression of NF-κB p65 by siRNA and found that p65 siRNA also reversed EMT and reduced migration and invasion abilities in GR cells. Therefore, our findings suggested that NF-κB p65 activation was vital to induce EMT phenotype and confer gefitinib resistance. NF-κB activation also plays critical roles in cancer development and progression [64], which makes it a therapeutic target for cancer therapy. Given that NF-κB also plays an important role in immune system, how to target it selectively in cancer is the major challenge [65].

To further investigate the molecular link or crosstalk between IGF1R and NF-κB p65, we first used IGF1 to activate IGF1R signaling in PC9 cells and found that NF-κB p65 was activated along with activation of Akt and Erk. We also treated PC9GR cells with IGF1R inhibitor PPP to inhibit the activated IGF1R and found that p65 was inhibited along with inhibition of Akt and Erk. These results indicated that IGF1R could activate p65 though Akt or Erk. Then we pre-treated PC9GR cells with Akt or Erk inhibitor to inhibit the activation of Akt or Erk and found that IGF1-induced p65 activation was blocked by either Akt or Erk inhibition, which further demonstrated that IGF1R activates Akt and Erk both of which could then activate p65. It is known that Akt or Erk could phosphorylate IKKα/β or directly phosphorylate p65, both resulting in p65 activation [66]. Therefore, we propose a model of how EGFR TKI-resistant EGFR-mutant NSCLC cells acquire EMT phenotype (Figure 5F). EGFR-mutant NSCLC cells capable of bypassing inhibited EGFR signaling through activated IGF1R are selected by EGFR TKI, i.e. GR cells. In GR cells, activated IGF1R activates Akt and Erk which could lead to NF-κB activation. As a result, NF-κB activation upregulates the expression of EMT transcription factors such as Snail, Slug or Zeb1 to induce EMT, which is also attributed to acquired EGFR TKI resistance.

In conclusion, we demonstrate for the first time that IGF1R/NF-κB pathway is involved in the acquisition of both EMT phenotype and EGFR TKI resistance in gefitinib-resistant EGFR-mutant NSCLC, suggesting that IGF1R/NF-κB pathway could be a novel therapeutic target for advanced NSCLC patients.

## MATERIALS AND METHODS

### Reagents

Gefitinib, parthenolide (PTL), LY294002 and U0126 were purchase from Selleck (Shanghai, China). Picropodophyllin (PPP) was purchased from Santa Cruz Biotechnology (Santa Cruz, CA, USA). Antibodies used for Western blotting were purchased from Cell Signaling Technology (Danvers, MA, USA) for Erk, phospho-p42/44 Erk (Thr202/ Tyr204), Akt, phospho-Akt (Ser473), IGF1R β, phospho-IGF1Rβ(Y1135, Y1135/1136), E-cadherin, vimentin, β-catenin, Snail, Zeb1, phospho-NF-κB p65 (Ser536), NF-κB p65, Histone H3 and β-actin. IGF1R β and NF-κB p65 siRNA were purchased from GenePharma (Shanghai, China). Horseradish peroxidase (HRP)-conjugated secondary antibody, AlexaFluor 488/594 conjugated secondary antibody, DAPI and Lipofectamine 3000 were purchased from Invitrogen (Carlsbad, CA, USA).

### Cell culture and establishment of gefitinib-resistant cell line

Human NSCLC HCC827 cells were purchased from the Cell Bank of Type Culture Collection of the Chinese Academy of Sciences (Shanghai, China) and PC9 cells were gifts from Dr. Qianggang Dong in Shanghai Cancer Institute, Shanghai Jiao Tong University School of Medicine. Both cell lines were routinely cultured in DMEM (Invitrogen, Carlsbad, CA, USA) supplemented with 10% fetal bovine serum (Invitrogen), 100μg/ml penicillin and 100 U/ml streptomycin sulfate in a humidified incubator at 37°C with 5% CO_2_. Gefitinib-resistant cells (PC9GR and HCC827GR) were established by stepwise escalation method: parental cells were cultured with stepwise escalation of concentration of gefitinib from 5nM/L to 5μM/L over 6 months.

### Western blot

Total cellular extracts were obtained by lysing cell pellets in RIPA buffer (50 mM Tris, pH7.4, 150 mM NaCl, 1% Triton X-100, 1% sodium deoxycholate, 0.1% SDS), while cytoplasmic extracts and nuclear extracts were prepared by using Nuclear and Cytoplasmic Protein Extraction Kit (Beyotime, Haimen, China) according to the manufacturer's instruction. Snap-frozen tumor nodules from lungs were homogenized in RIPA buffer on ice and supernatant was collected after centrifuged at 13,000 g for 10 min at 4°C. All lysis buffers were supplemented with protease inhibitor (Sigma-Aldrich) and phosphatase inhibitors (Sangon Biotech, Shanghai, China). Equal amounts of proteins were separated on 10% SDS-PAGE and then transferred to PVDF membranes (Millipore, Billerica, MA, USA). Membranes were blocked with PBS buffer containing 5% non-fat milk and 0.1% Tween 20 (PBST) and then incubated with primary antibodies (1:500-1:1000) overnight at 4°C. After being washed three times with PBST, membranes were incubated with peroxidase-conjugated secondary antibodies (1:3000) for 1 h and then washed with PBST again and developed with ECL (Pierce, Rockford, IL, USA). β-actin was used as an internal control for loading control.

### Cell viability assay

The proliferative capacity of cells was assessed using the cell counting kit-8 (CCK8) (Dojindo, Shanghai, China) according to the manufacturer's instructions as described previously [51]. Briefly, cells were seeded at a density of 3000-5000 cells/well in 96-well plates. After incubated with serum-free DMEM for 24 h, the cells were treated with indicated concentrations of gefitinib for 72h. Cells treated with solvent (DMSO) were used as a control, with viability set at 100%.

### siRNA transfection

Cells were seeded in six-well plates and transfected with gene-specific siRNA or control siRNA using Lipofectamine 3000 as described previously. The cells were subjected to further experiments after 48h after transfection. The sequences used for NF-κB p65 siRNA sequences are as following: 5′-GCCCUAUCCCUUUACGUCA-3′ and 5′-UGACGUAAAGGGAUAGGGC-3′. The sequences used for IGF1Rβ siRNA sequences are as following: 5′-CGACUAUCAGCAGCUGAAG-3′ and 5′-CUUCAG CUGCUGAUAGUCG-3′.

### Wound healing assay

Cells were allowed to grow to 90% confluency in 6-well flat-bottomed plates and serum starved for 24 h. After aspirating the medium, the monolayer was scratched using a 200μL pipette tip. The cells were washed with PBS to remove detached cells and then incubated in medium containing 1% FBS. The scratched areas were photographed at 0, 24, 48 and 72h after scratching using microscopy. Cell migration was calculated as percentages of open wound area to the initial blank areas containing no cells using ImageJ software. The values are the means of three independent experiments.

### Transwell migration and invasion assays

Migration assay was performed in 24-well inserts (8 μm pore size; Corning Inc, Corning, NY, USA) and invasion assay was performed in 24-well Matrigel invasion inserts (8 μm pore size; Corning Inc, Corning, NY, USA) according to manufacturer's instructions. 1-2×10^4^ cells in serum-free DMEM were seeded in the upper chamber of the insert and DMEM containing 10% fetal bovine serum was added to the lower chamber. Cells were incubated at 37°C in 5% CO_2_ for 8h (for migration) or 24h (for invasion). Non-migrated cells were scraped from the upper surface of the membrane with a cotton swab, and migrated cells remaining on the bottom surface were fixed with 10% paraformaldehyde for 20 min, stained with a 0.1% crystal violet solution for 2 hours, and then photographed and counted under a microscopy from at least 7 randomly selected fields.

### Immunofluorescence and confocal microscopy

Cells were fixed in 4% paraformaldehyde, permeabilized in 0.2% Triton X-100, blocked in 1% BSA, and then stained with antibody (1:100) at 4°C overnight. After being washed three times with PBS, cells were incubated with FITC-conjugated secondary antibodies (1:100) for 1h, and then washed with PBS again and incubated with Hoest 33342 for nuclear counterstaining. Cells were visualized with a Zeiss LSM710 (Zeiss, Thornwood, NY, USA) confocal microscope.

### Genetically engineered mice studies

The double-transgenic mice (Tet-op-hEGFR Del19-Luc and CCSP-rtTA) were generously provided by Dr. Kin-Kong Wong at Dana-Farber Cancer Institute, Harvard Medical School [49]. The mice were housed in pathogen-free environment at Shanghai Jiao Tong University School of Medicine. All experimental procedures were reviewed and approved in accordance with the guidelines for the care and use of laboratory animals at Shanghai Jiao Tong University. As previously described [49], the double-transgenic mice were exposed to a doxycycline-containing diet for 4 to 6 weeks and then subjected to treatment with gefitinib formulated in 0.5% methocellulose-0.4% polysorbate-80 (Tween 80) by gavage at 50 mg/kg daily for two weeks and then at 25 mg/kg every other day for at least 4-6 month until persistent symptoms of respiratory distress emerged. The littermate control mice were treated with vehicle for one week.

### Statistical analysis

All data are presented as the mean±SEM. Statistical analysis was conducted using GraphPad Prism 5.0 software (La Jolla, CA, USA). Differences between groups were examined using Student's t-test. Differences were considered significant if P value was less than 0.05.

## SUPPLEMENTARY MATERIALS FIGURES



## Abbreviations

NSCLC: Non-small cell lung cancer
EGFR: Epidermal growth factor receptor
TKI: Tyrosine kinase inhibitor
EMT: Epithelial-mesenchymal transition
IGF1: Insulin-like growth factor 1
IGF1R: Insulin-like growth factor 1 receptor
GR: Gefitinib-resistant
NF-κB: Nuclear factor kappa-light-chain-enhancer of activated B cells
IKKα/β: Inhibitor of nuclear factor kappa-B kinase subunit α/β
PPP: Picropodophyllin
PTL: Parthenolide
