# Supplementary material for: Acquisition of EGFR TKI resistance and EMT phenotype is linked with activation of IGF1R/NF-κB pathway in EGFR-mutant NSCLC
Source: Oncotarget. 2017 Sep 21;8(54):92240–53. doi: 10.18632/oncotarget.21170 (PMC5696177; doi:10.18632/oncotarget.21170)
Supplement: Supplementary file 1 [file oncotarget-08-92240-s001.pdf]

Acquisition of EGFR TKI resistance and EMT phenotype is linked with activation of IGF1R/NF-κB pathway in EGFR-mutant NSCLC

SUPPLEMENTARY MATERIALS

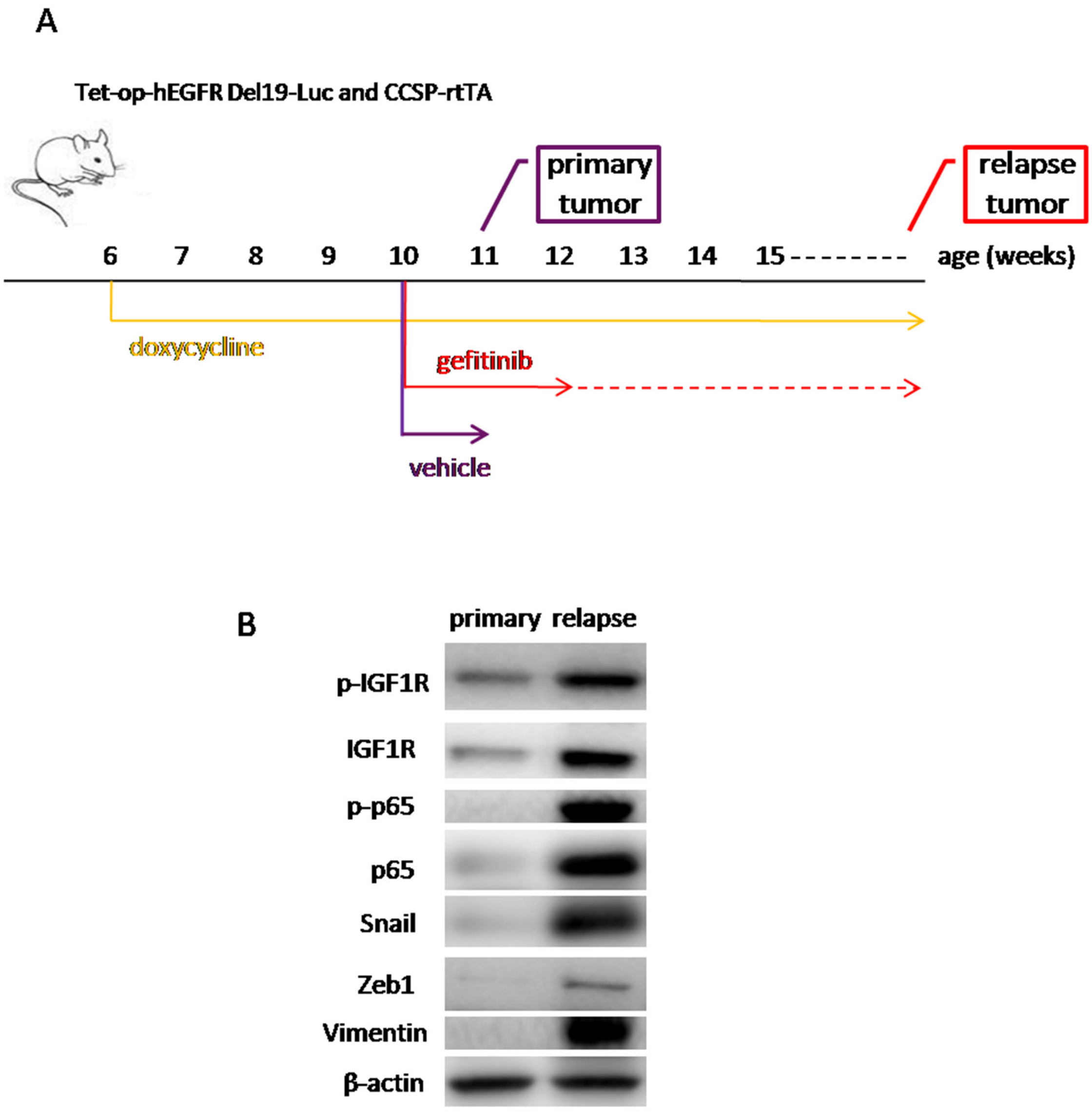

Supplementary Figure 1: IGF1R/NF-κB p65 signaling was activated in gefitinib-resistant mouse lung adenocarcinoma. **(A)** Line chart depicting the schedule used to treat transgenic mice (Tet-op-hEGFR Del19-Luc and CCSP-rtTA) with gefitinib. Doxycycline administration was initiated at the age of 6 weeks and subsequently kept constant throughout the life of the animal (yellow line indicates continuous administration of doxycycline until sacrificed). Mice were treated with gefitinib daily for 2 weeks (red solid line indicates treatment with 50 mg/kg gefitinib daily for 2 weeks) and then every other day for 4-6 months until persistent symptoms of respiratory distress emerged (red dotted line indicates treatment with 25 mg/kg gefitinib every other day). The littermate control mice were treated with vehicle for one week (purple line indicates treatment with vehicle for 1 week). **(B)** The expression of IGF1R, p-IGF1R, p65, p-p65, Snail, Zeb1 and vimentin of lung tumors isolated from transgenic mouse (Tet-op-hEGFR Del19-Luc and CCSP-rtTA) treated with gefitinib for 4-6 months (relapse tumor) and the littermate control mouse treated with vehicle (primary tumor) was examined by Western blotting.

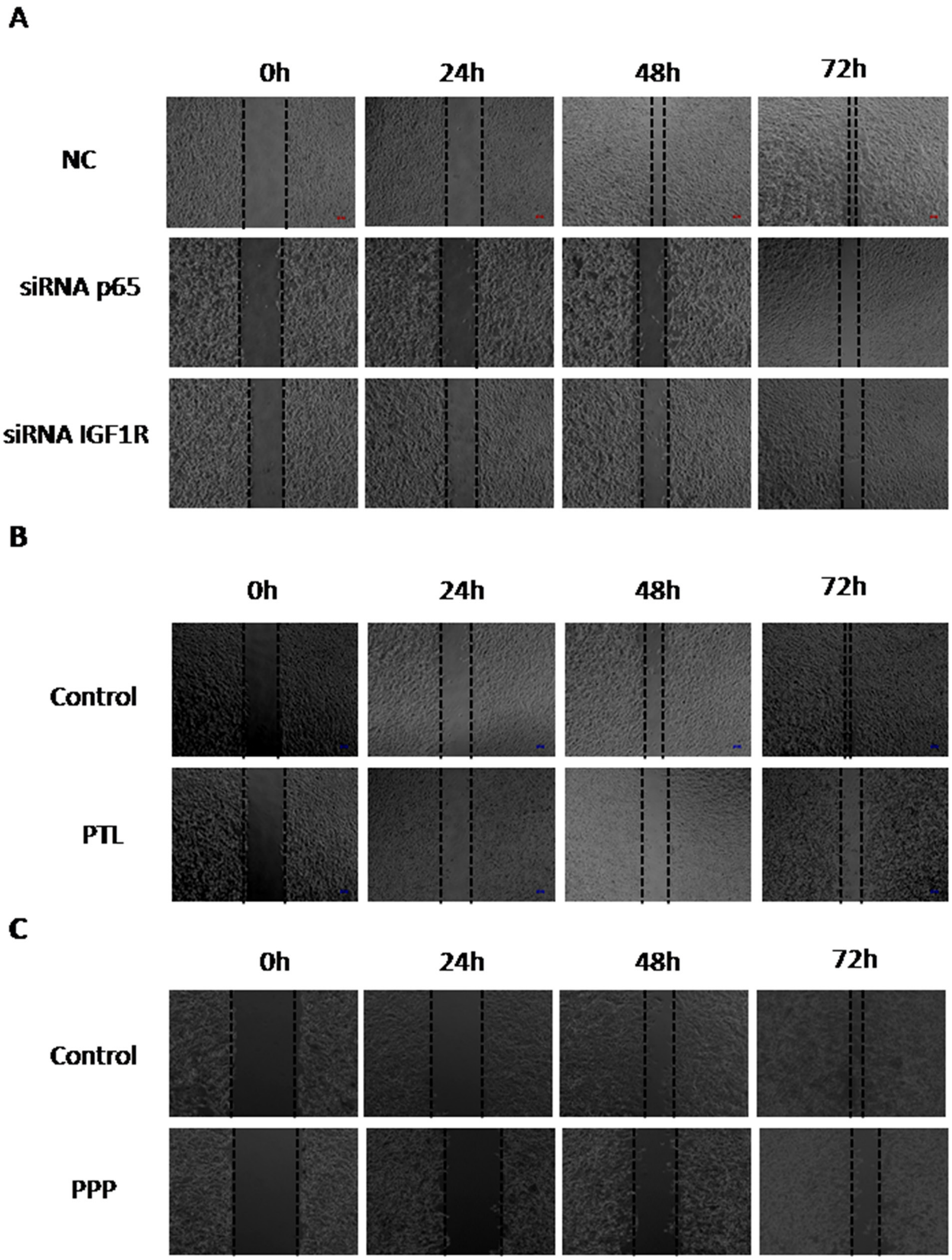

Supplementary Figure 2: Inhibition of IGF1R or NF-κB p65 suppressed migration in PC9GR cells. (A) Wound healing assay of PC9GR cells transfected with siRNA p65 or siRNA IGF1R. **(B)** Wound healing assay of PC9GR cells treated with 1μM PTL. **(C)** Wound healing assay of PC9GR cells treated with 0.1μM PPP. Images were taken at 0, 24, 48 and 72h with a 4× objective lens. Scale bar: 100μm. The black dotted lines depict the area of wound closure.

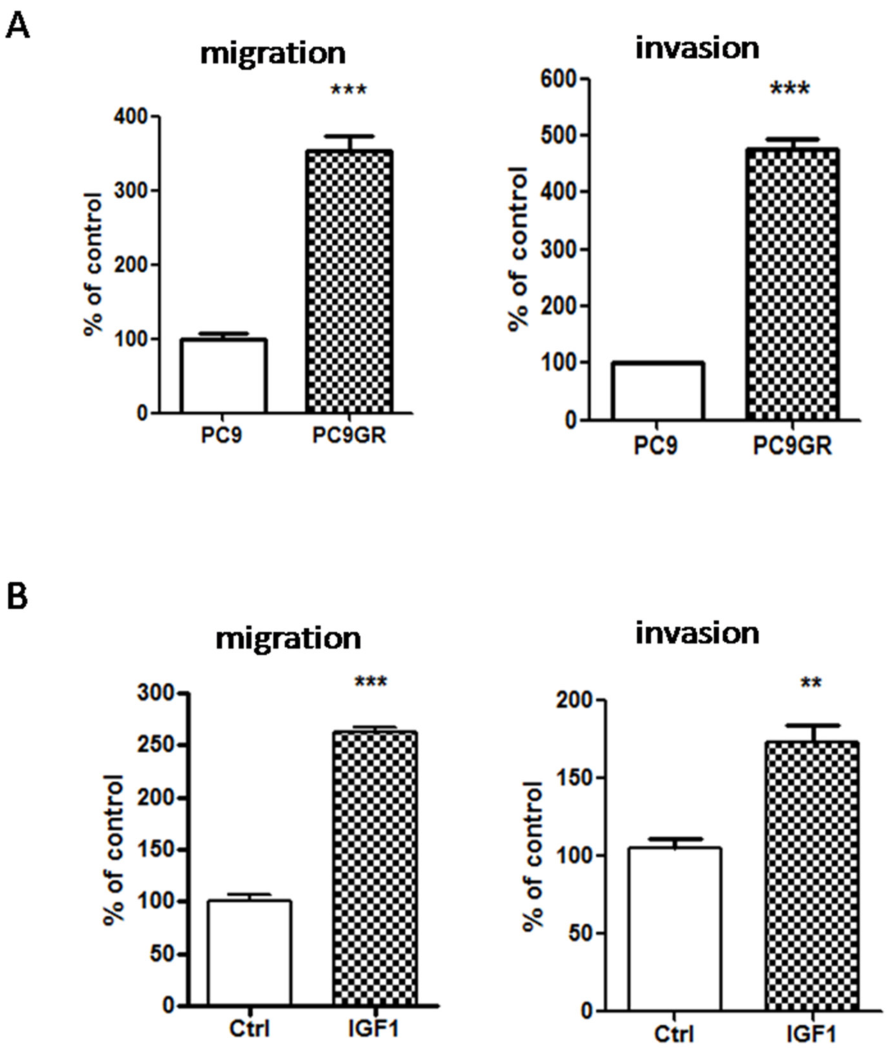

Supplementary Figure 3: Quantification of transwell migration and invasion assays. (A) Quantification of Figure 1E. The number of cells was counted from at least four independent microscopic fields. *** P < 0.001, compared with PC9. **(B)** Quantification of Figure 5B. ** P < 0.01; *** P < 0.001, compared with control.
